# Primary Renal Leiomyosarcoma: A Case Report of a Rare and Aggressive Neoplasm

**DOI:** 10.7759/cureus.87099

**Published:** 2025-07-01

**Authors:** Srinidhi Srinivasan, Arun Paul, Hima Pravallika, Indra Neil Mekala, Venkateswar Rao Sriramaneni

**Affiliations:** 1 Radiodiagnosis, Alluri Sitarama Raju Academy of Medical Sciences College and Hospital, Eluru, IND

**Keywords:** inferior vena cava tumor thrombus, kidney leiomyosarcoma, pleural metastasis, renal cell carcinoma (rcc), smooth muscle actin (sma)

## Abstract

Primary sarcomas of renal origin are among the rarest tumors to present, and definitive diagnosis is based primarily on histopathology since there are no characteristic clinical features. Renal sarcomas are known for their aggressive growth and late presentation, resulting in a particularly poor prognosis. This article reports a case of primary leiomyosarcoma of renal origin in a 38-year-old female patient who presented with complaints of pain and a palpable abdominal mass. A contrast-enhanced computed tomography (CECT) scan of the abdomen revealed a large tumor in the left kidney and tumor thrombus in the left renal vein extending up to the intrahepatic portion of the inferior vena cava (IVC). The patient underwent radical nephrectomy, and histopathological examination confirmed the diagnosis of renal leiomyosarcoma.

## Introduction

Renal cell carcinomas (RCC) are considered the most common malignant renal tumors. However, other varieties of renal tumors occur in the renal parenchyma and mimic RCC on cross-sectional imaging studies. Renal leiomyosarcomas are extremely rare solid neoplasms of the kidney, accounting for only 0.1% of all invasive renal tumors [1]. They present more often in females during the fifth to sixth decades of life [2,3]. Due to their nonspecific clinical symptoms and radiological findings, preoperative diagnosis of this tumor subtype is challenging. Renal leiomyosarcomas are notorious for their propensity for hematogenous metastasis to distant body organs [4].

## Case presentation

A 38-year-old female patient was referred to the radiology department from surgical oncology with complaints of pain in the left loin region and a palpable swelling in the left hypochondrium. The mass showed rapid growth over the past two months, accompanied by weight loss and increased frequency of micturition over the past year. The patient had a known history of type 2 diabetes mellitus and hypertension and was on regular medication.

**Figure 1:**
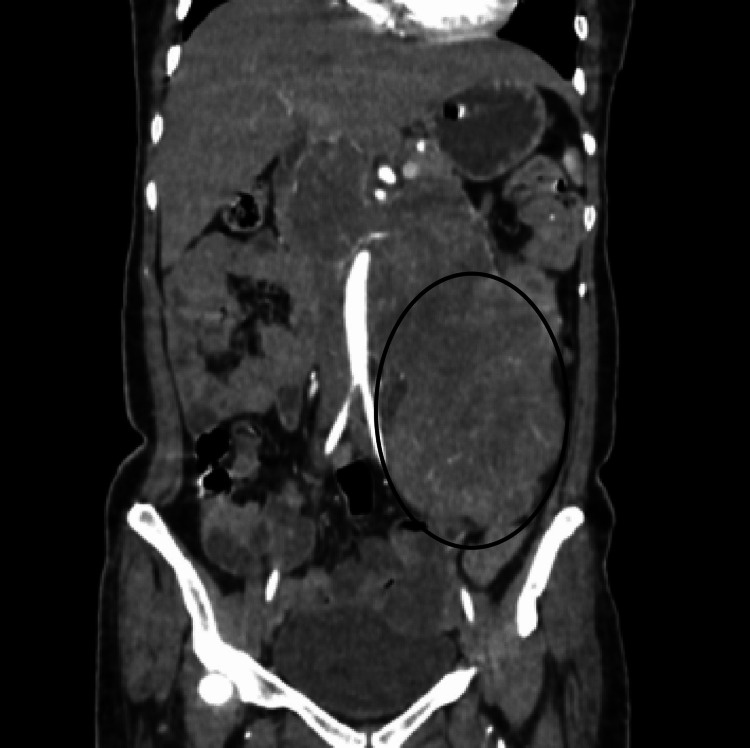
CECT abdomen coronal section showing a well-defined, lobulated, heterogeneously enhancing (circled) mass lesion involving almost the entire left kidney but sparing the upper pole. The lesion shows areas of necrosis. CECT: contrast-enhanced computed tomography

A contrast-enhanced computed tomography (CECT) scan of the abdomen revealed a 17 × 15.5 × 10 cm heterogeneous mass lesion involving almost the entire left kidney but sparing the upper pole (Figure 1). The lesion was seen extending up to the abdominal wall anteroposteriorly with displacement of adjacent bowel loops. The post-contrast study showed heterogeneous enhancement with central non-enhancing areas, suggestive of necrosis. However, unlike the classic ball-type shape that is observed in renal cell carcinoma, the lesion was oblong in shape due to its large size.

**Figure 2:**
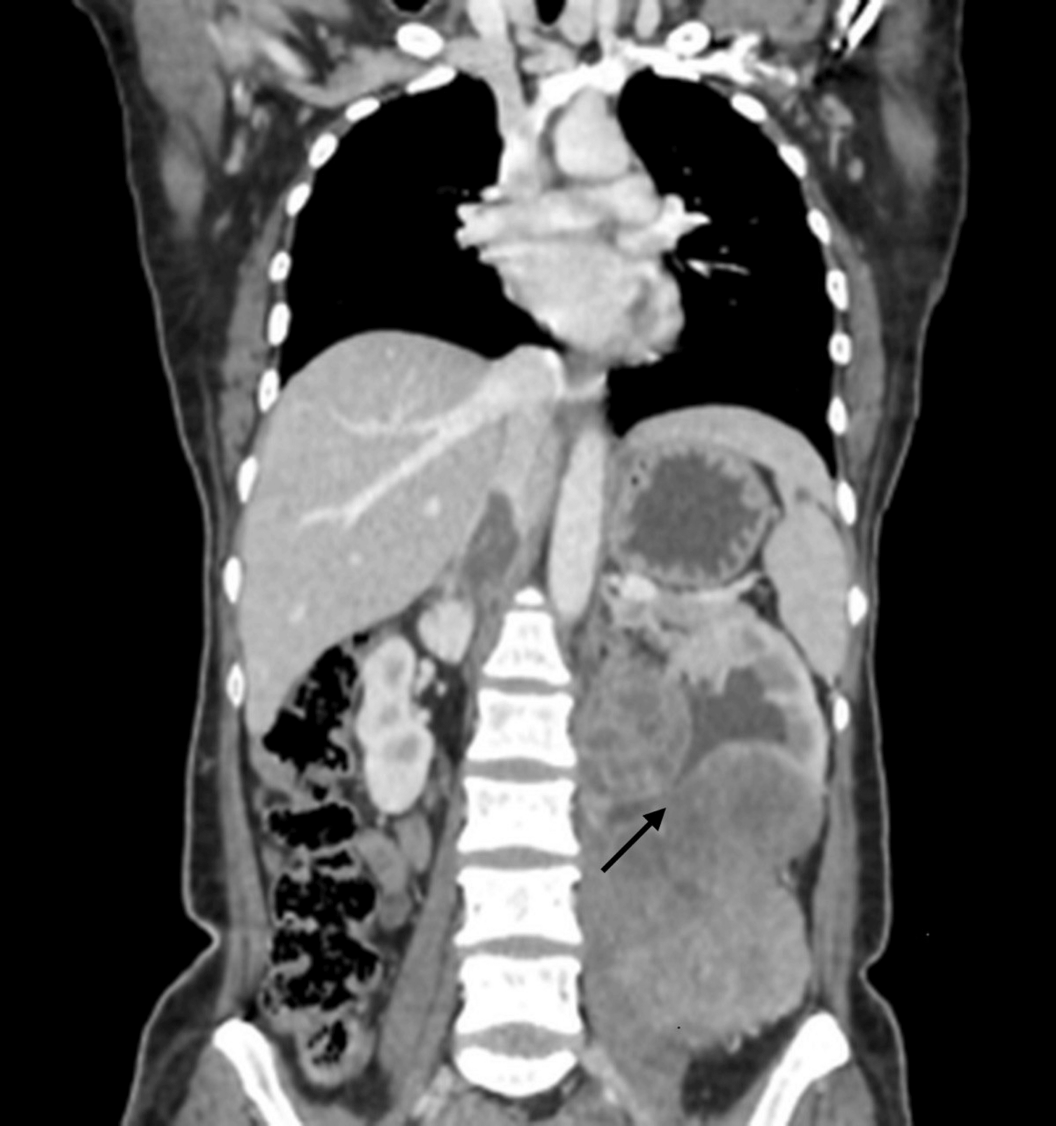
CECT abdomen coronal section showing encasement of the left proximal ureter by the lesion (arrow), causing moderate hydroureteronephrosis. CECT: contrast-enhanced computed tomography

**Figure 3:**
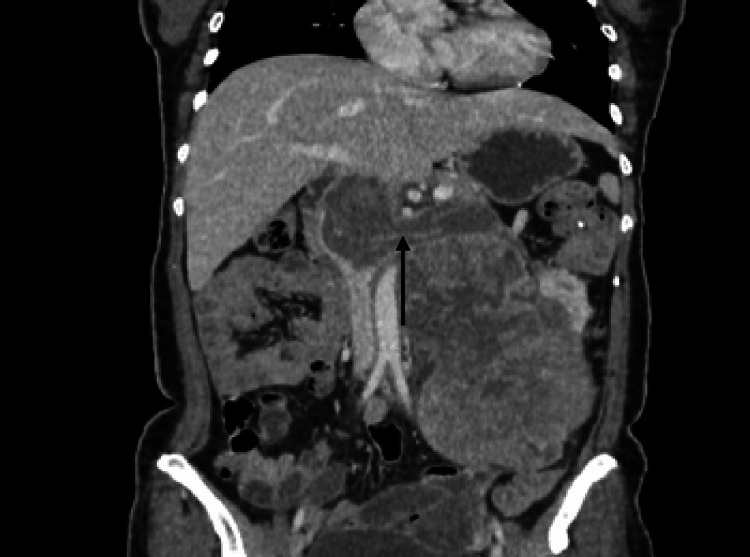
CECT abdomen coronal section showing extension of tumor thrombus from the left renal vein into the IVC. CECT: contrast-enhanced computed tomography; IVC: inferior vena cava

**Figure 4:**
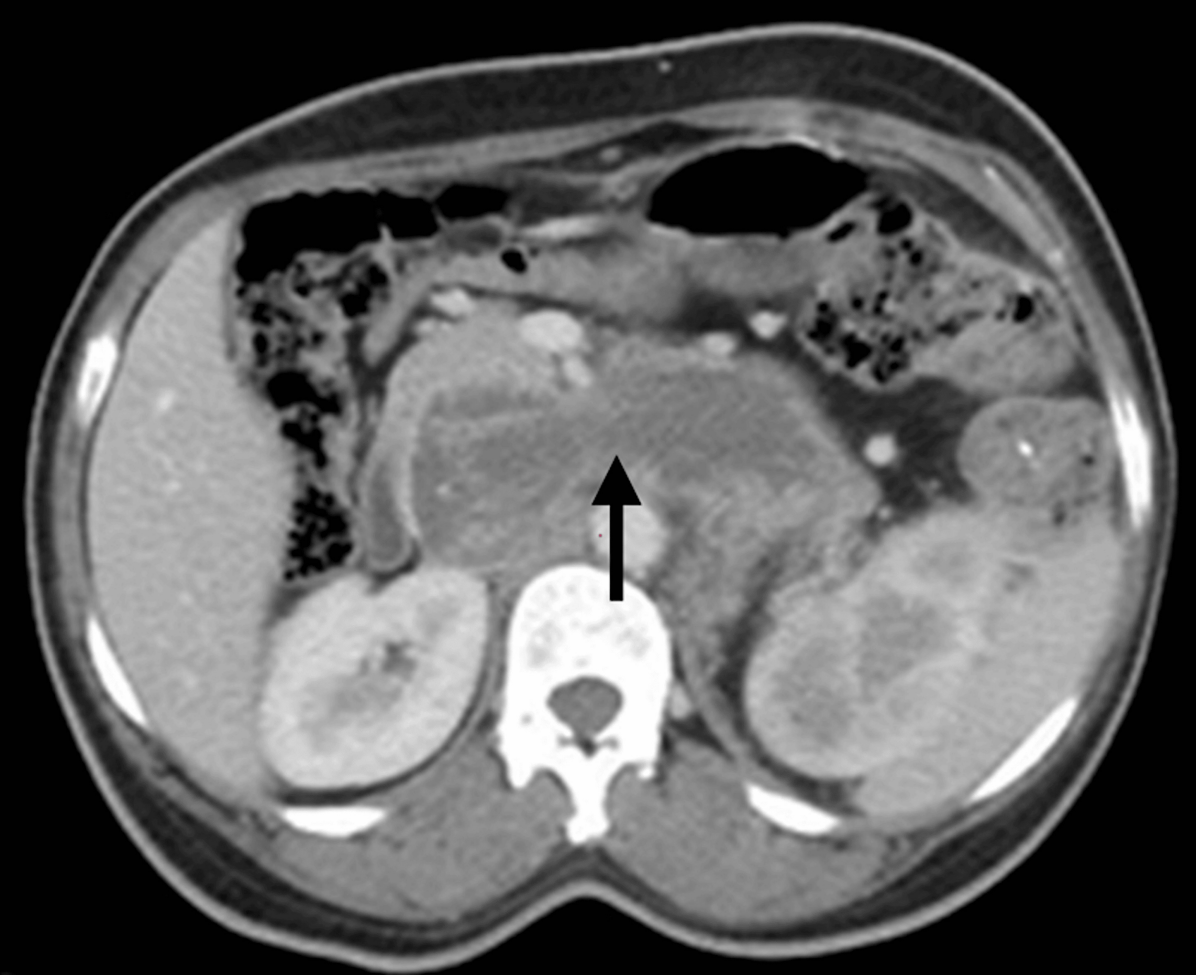
CECT abdomen axial section showing extension of tumor thrombus from the left renal vein into the IVC. CECT: contrast-enhanced computed tomography; IVC: inferior vena cava

**Figure 5:**
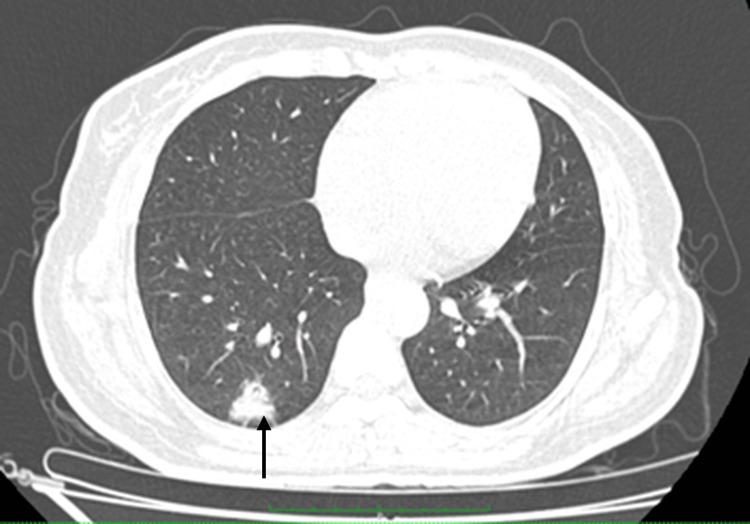
CECT axial section showing an enhancing nodule with spiculated margins (arrow) noted in the posterobasal segment of the right lower lobe, suspicious of a malignant nodule. CECT: contrast-enhanced computed tomography

There was encasement of the left proximal ureter by the lesion, causing moderate hydroureteronephrosis (Figure 2). The left renal vein showed a tumor thrombus that was extending up to the intrahepatic portion of the inferior vena cava (IVC), which was shown in both axial (Figure 3) and coronal (Figure 4) sections. This tumor thrombus was seen displacing the pancreas anteriorly. A solitary pulmonary nodule was noted in the posterobasal segment of the left lower lobe, possibly of metastatic origin (Figure 5).

**Figure 6:**
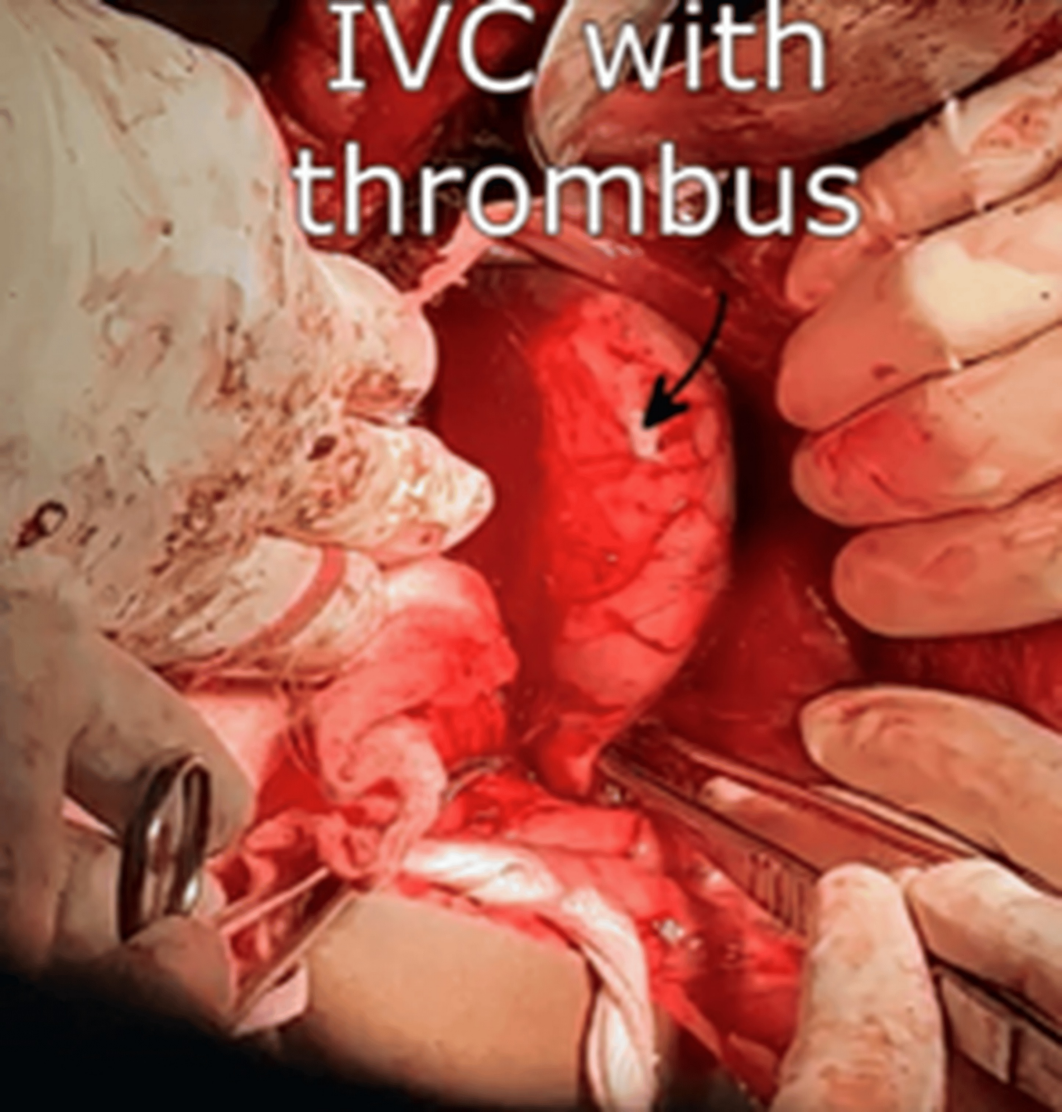
Intraoperative findings revealing an IVC with thrombus. IVC: inferior vena cava

**Figure 7:**
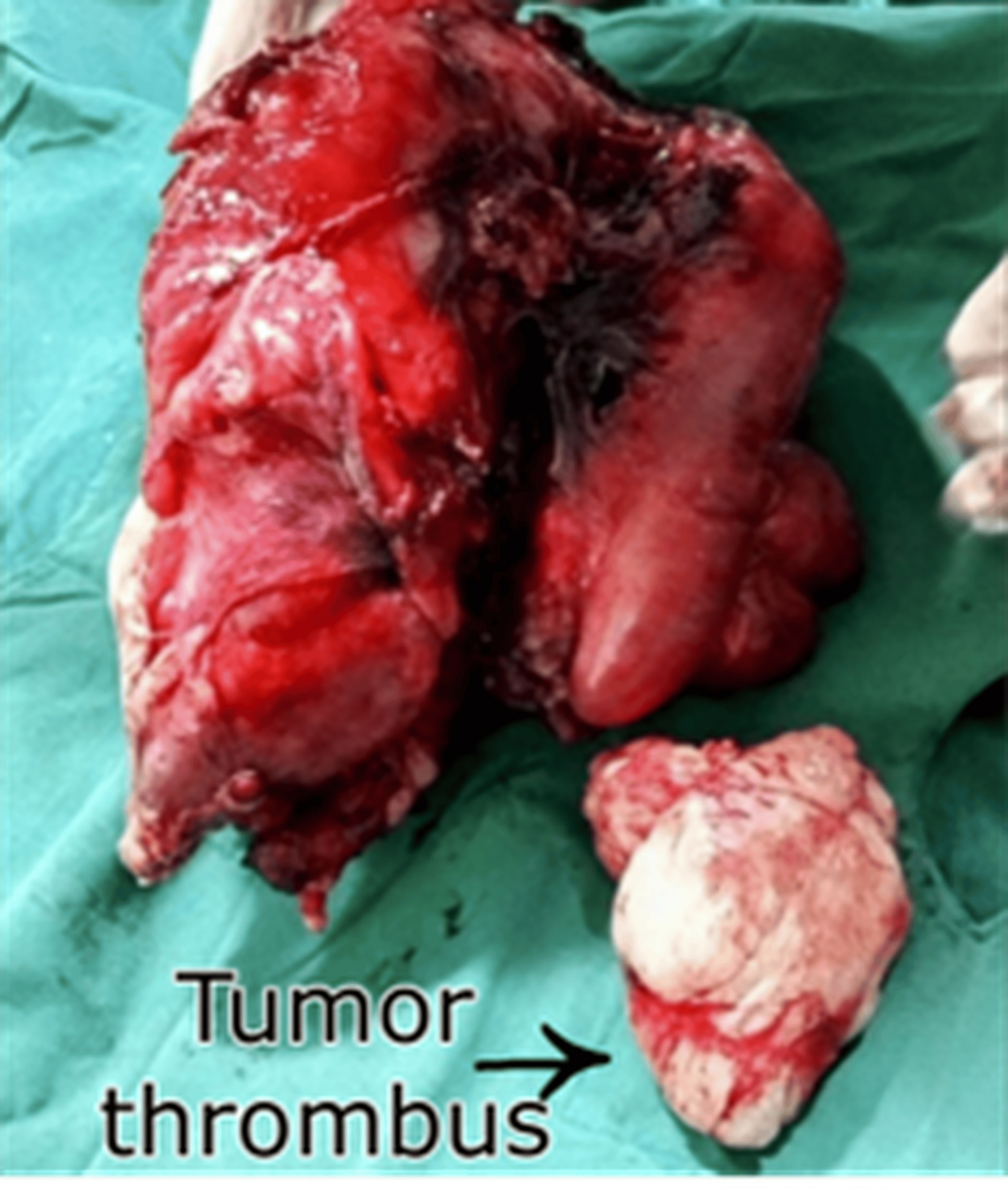
Gross specimen appearing as a large, bosselated, grayish-white mass with a fish-flesh appearance, showing areas of necrosis and hemorrhage. A separate specimen (arrow) showing a tumor thrombus.

Radical left nephrectomy with removal of the IVC thrombus (Figure 6) was performed, and the specimen was sent for histopathological examination (HPE). The specimen appeared as a large, bosselated, grayish-white mass showing areas of hemorrhage and necrosis. It occupied the entire left kidney, sparing a thin rim of the upper one-third of the renal parenchyma. A separate specimen revealed grayish-brown friable tissue from the IVC tumor thrombus (Figure 7).

**Figure 8:**
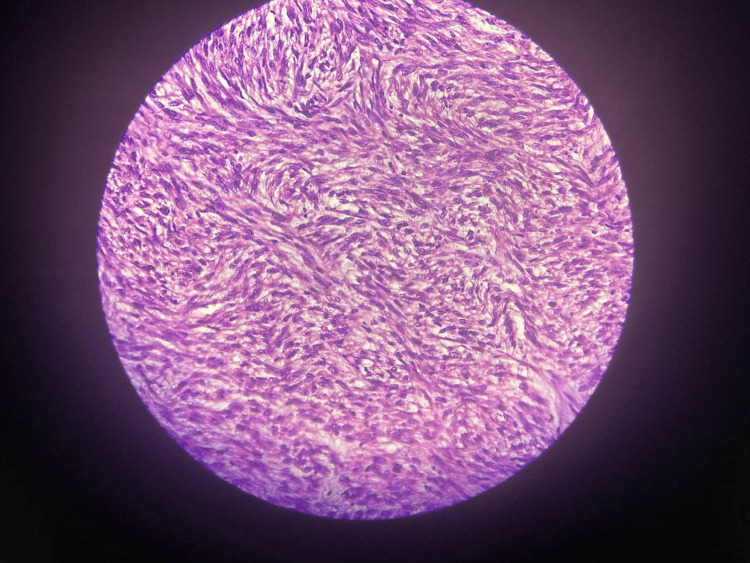
Histopathological examination revealed interlacing bundles of muscle fascicles with spindle cells exhibiting cigar-shaped nuclei.

**Figure 9:**
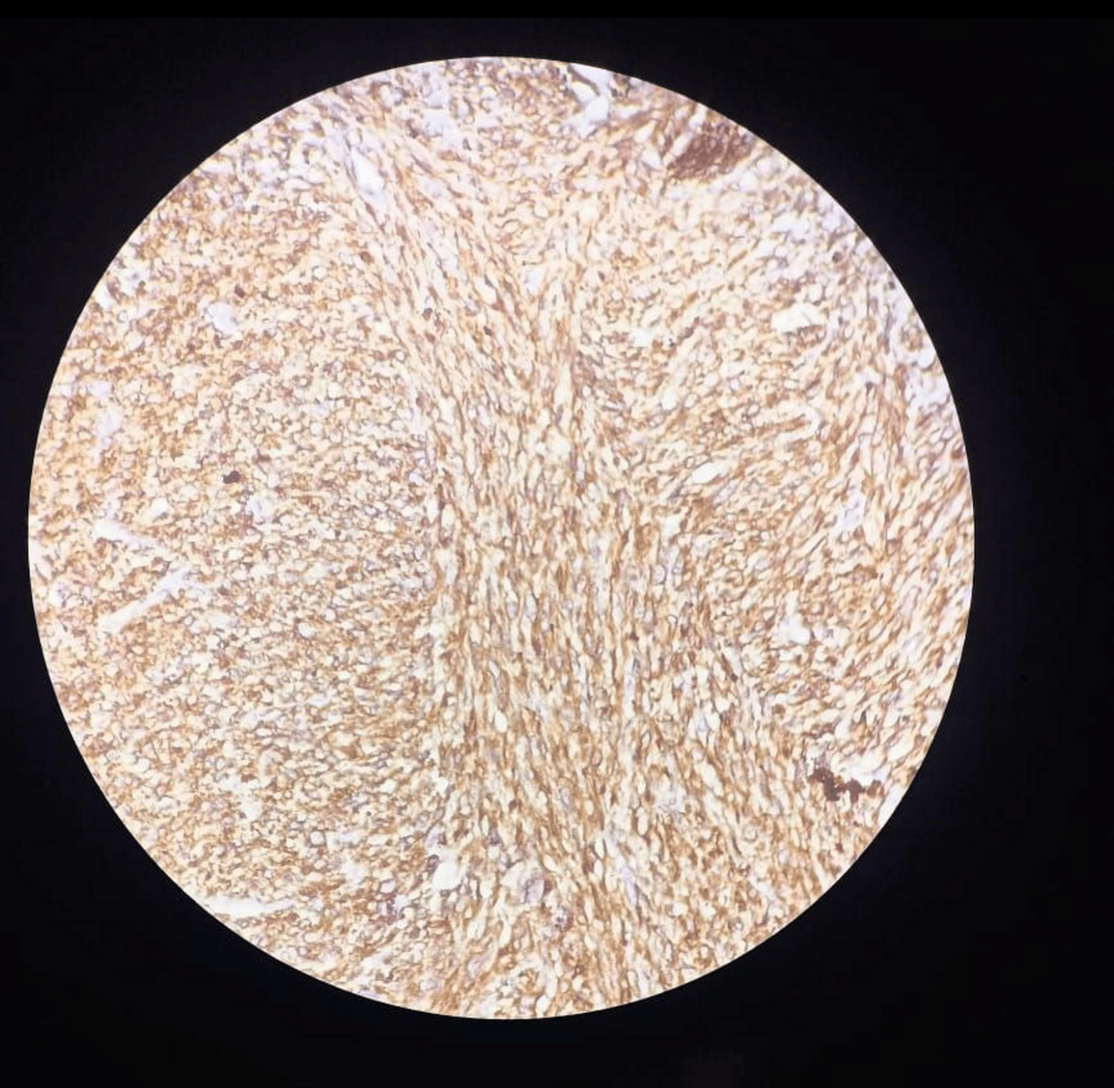
SMA positive on IHC examination. IHC: immunohistochemistry; SMA: smooth muscle actin

**Figure 10:**
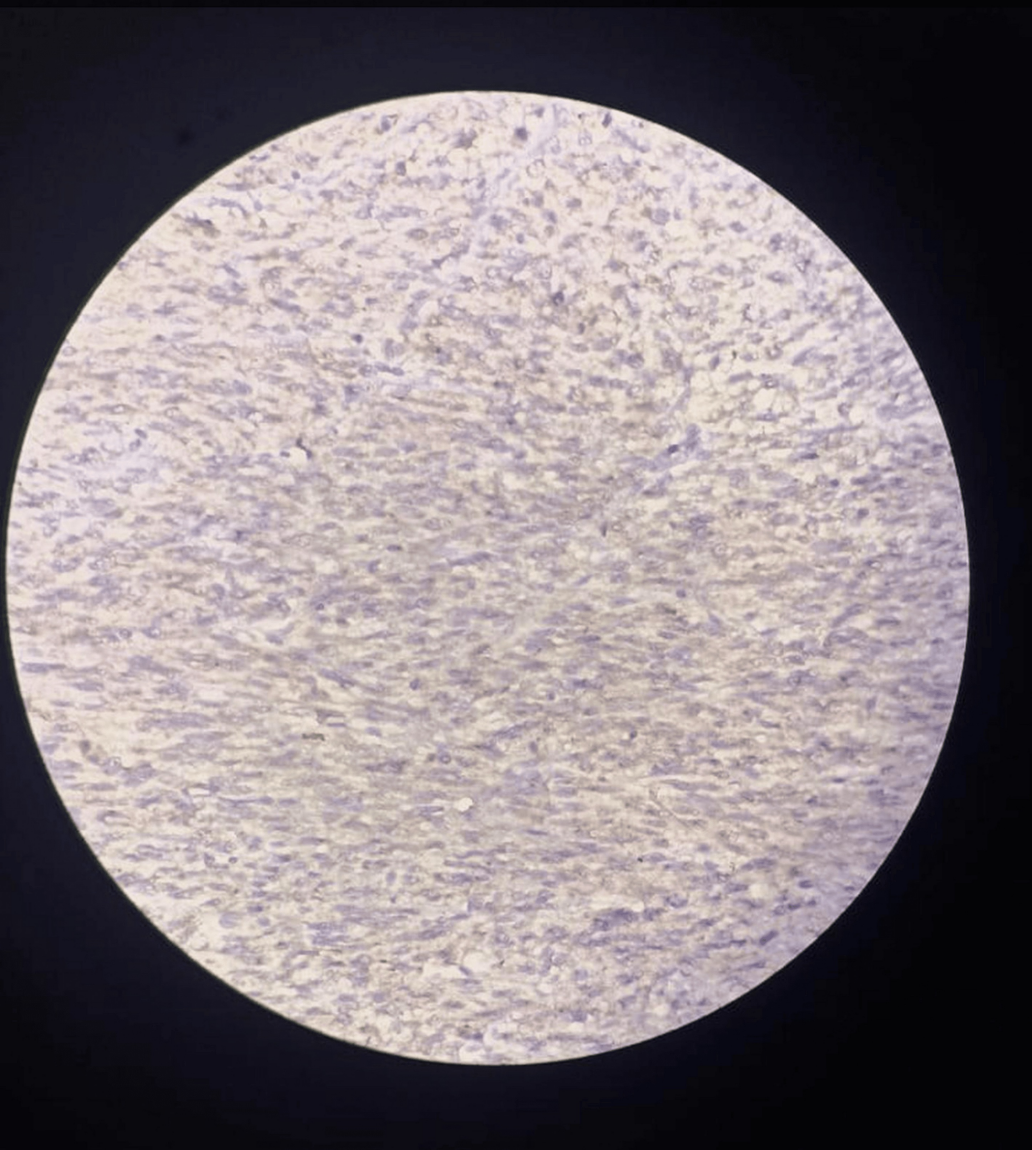
Cytokeratin negative on IHC examination. IHC: immunohistochemistry

Histopathology revealed interlacing bundles of muscle fascicles with spindle cells exhibiting cigar-shaped nuclei, eosinophilic cytoplasm, cellular pleomorphism, and foci of tumor necrosis, consistent with renal leiomyosarcoma (Figure 8). On immunohistochemistry, the specimen was found to be smooth muscle actin (SMA) positive (Figure 9) and cytokeratin (CK) negative (Figure 10). However, further follow-up examinations could not be done due to the patient's financial constraints and limited diagnostic resources.

## Discussion

Renal leiomyosarcomas represent 50-60% of all renal sarcomas, yet they remain exceedingly rare tumors. They can arise from the smooth muscle fibers of the renal parenchyma, renal capsule, renal pelvis, or renal vessels [1]. In a systematic review conducted by Periasamy et al., the clinical presentation for primary renal leiomyosarcoma included abdominal pain, abdominal mass, hematuria, or a combination of these, thus mimicking other renal tumors. Tumor size >5 cm was found in more than 50% of patients at the time of diagnosis due to the lack of natural barriers, with emergency presentations of spontaneous rupture reported [5].

On computed tomography, they appear as well-circumscribed, exophytic, solid masses that show heterogeneous enhancement, and when large, they present as multilocular cystic masses with peripheral enhancement [1]. Additionally, approximately 30% of these lesions demonstrate varying degrees of calcification [6].

These exhibit characteristics similar to leiomyosarcomas found elsewhere in the body. Histopathological examination reveals interweaving bundles of muscle fibers with spindle-shaped cells, characterized by increased cellularity, cellular irregularity, cigar-shaped nuclei, eosinophilic cytoplasm, and areas of tumor necrosis. Leiomyosarcoma tumor cells typically show positive staining for SMA, desmin, calponin, and h-caldesmon and negative staining for CK, S-100 protein, HMB-45, and CD117 (c-KIT) [7-9].

Unfortunately, 90% of patients develop distant metastases, and the disease proves fatal for 75% of those affected [6,10]. The presence of a tumor thrombus in the IVC increases the risk of tumor spread [6,11]. Currently, surgical removal is the only potentially curative treatment for resectable leiomyosarcomas, with palliative chemotherapy or radiation recommended in some studies, although some studies have reported that there is no significant benefit noted from radiation [12]. Due to the high risk of local recurrence, radical nephrectomy, which involves removing the entire kidney, is considered a more effective treatment option, offering better cancer control and outcomes [13].

Previous studies have shown that renal leiomyosarcoma typically presents in the fifth to sixth decades of life [13]. However, in our case, a middle-aged female in her fourth decade presented with an aggressive renal mass with tumor thrombus in the renal vein and IVC. Hence, leiomyosarcoma should also be considered alongside renal cell carcinoma when evaluating such aggressive lesions in younger patients [3]. Additionally, our case had a possible metastatic nodule in the left lung, which implies that a simultaneous screening of the chest and follow-up with positron emission tomography/computed tomography (PET/CT) would provide valuable additional information regarding distant metastasis to the lung, particularly in cases where tumor thrombus is noted in the IVC, if sufficient diagnostic resources are available [14]. Unfortunately, further follow-up investigations could not be done for our case due to the patient's financial constraints and limited diagnostic resources. Nevertheless, in the given setting, it is most likely to have originated from primary renal malignancy.

## Conclusions

Leiomyosarcomas of renal origin are extremely rare tumors. This case highlights the importance of considering leiomyosarcoma in addition to renal cell carcinoma in the differential diagnosis of aggressive renal lesions in middle-aged females. Analyzing the presenting features, imaging findings, and histopathological features of these tumors helps clinicians identify these subtypes for accurate diagnosis and treatment.
